# Chronically Activated T-cells Retain Their Inflammatory Properties in Common Variable Immunodeficiency

**DOI:** 10.1007/s10875-021-01084-6

**Published:** 2021-07-11

**Authors:** Roos-Marijn Berbers, M. Marlot van der Wal, Joris M. van Montfrans, Pauline M. Ellerbroek, Virgil A. S. H. Dalm, P. Martin van Hagen, Helen L. Leavis, Femke van Wijk

**Affiliations:** 1 Department of Rheumatology and Clinical Immunology, University Medical Center and Utrecht University, Utrecht, The Netherlands; 2 Center for Translational Immunology, University Medical Center Utrecht and Utrecht University, Utrecht, The Netherlands; 3 Department of Pediatric Immunology and Infectious Diseases, University Medical Center Utrecht and Utrecht University, Utrecht, The Netherlands; 4 Department of Internal Medicine and Infectious Diseases, University Medical Center Utrecht and Utrecht University, Utrecht, The Netherlands; 5 Department of Internal Medicine, Division of Clinical Immunology, Erasmus MC University Medical Center, Rotterdam, The Netherlands; 6 Department of Immunology, Academic Center for Rare Immunological Diseases (RIDC), Erasmus MC University Medical Center, Rotterdam, The Netherlands

**Keywords:** Immune dysregulation, Common variable immunodeficiency (CVID), T-cells, Immune exhaustion, Regulatory T-cells, Autoimmunity

## Abstract

**Purpose:**

Immune dysregulation complications cause significant morbidity and mortality in common variable immunodeficiency (CVID), but the underlying pathophysiology is poorly understood. While CVID is primarily considered a B-cell defect, resulting in the characteristic hypogammaglobulinemia, T-cells may also contribute to immune dysregulation complications. Here, we aim to further characterize T-cell activation and regulation in CVID with immune dysregulation (CVIDid).

**Methods:**

Flow cytometry was performed to investigate T-cell differentiation, activation and intracellular cytokine production, negative regulators of immune activation, regulatory T-cells (Treg), and homing markers in 12 healthy controls, 12 CVID patients with infections only (CVIDio), and 20 CVIDid patients.

**Results:**

Both CD4 + and CD8 + T-cells in CVIDid showed an increased activation profile (HLA-DR + , Ki67 + , IFNγ +) when compared to CVIDio, with concomitant upregulation of negative regulators of immune activation PD1, LAG3, CTLA4, and TIGIT. PD1 + and LAG3 + subpopulations contained equal or increased frequencies of cells with the capacity to produce IFNγ, Ki67, and/or GzmB. The expression of PD1 correlated with serum levels of CXCL9, 10, and 11. Treg frequencies were normal to high in CVIDid, but CVIDid Tregs had reduced CTLA-4 expression, especially on CD27 + effector Tregs. Increased migratory capacity to inflamed and mucosal tissue was also observed in CVIDid T-cells.

**Conclusion:**

CVIDid was characterized by chronic activation of peripheral T-cells with preserved inflammatory potential rather than functional exhaustion, and increased tissue migratory capacity. While Treg numbers were normal in CVIDid Tregs, low levels of CTLA-4 indicate possible Treg dysfunction. Combined studies of T-cell dysfunction and circulating inflammatory proteins may direct future treatment strategies.

**Supplementary Information:**

The online version contains supplementary material available at 10.1007/s10875-021-01084-6.

## Introduction

Common variable immunodeficiency (CVID) is characterized by recurrent infections caused by low IgG, and IgA or IgM [1], for which patients are treated with immunoglobulin G replacement therapy (IgRT) [2]. IgRT has significantly decreased the risk of infectious complications in CVID, but nonetheless, over a third of patients develop immune dysregulation complications [3, 4], resulting in significant morbidity and mortality [5, 6]. A wide range of immune dysregulation phenomena can be observed in CVID, including granulomatous-lymphocytic interstitial lung disease (GLILD), enteritis, autoimmune cytopenias, lymphoproliferation, and hematological malignancies [4, 7]. The underlying pathophysiology of immune dysregulation in CVID is currently poorly understood, which complicates diagnostics and treatment [8, 9].

While the defining hypogammaglobinemia in CVID is considered to be primarily the result of B-cell dysfunction, several lines of evidence suggest an additional role for T-cells in CVID with immune dysregulation (CVIDid). Biopsies of lung granulomas in CVIDid show predominance of CD4 + T helper (Th) cells [10, 11], while regulatory T-cells (Tregs) are often absent [10]. In peripheral blood of patients with CVIDid, a decreased CD4/CD8 ratio was observed with decrease of naïve T-cells [12], Tregs, Th17 cells, and follicular helper T (Tfh) cells [13, 14]. Moreover, there are indications that CVID T-cells may be functionally exhausted, including reduced capacity to respond to bacterial antigens and increased expression of PD1 [15, 16]. Our group and others have previously demonstrated that serum cytokines in CVIDid [17, 18] are shifted towards a Th1 phenotype, and we observed an upregulation of proteins associated with immune regulation—IL10, LAG3, and 4-1BB. Monogenic primary immunodeficiencies caused by mutations in immune regulation genes such as CTLA4 [19] and ICOS [20] often result in a CVIDid phenotype. However, how the interplay between immune regulation and immune activation results in CVIDid remains poorly understood.

To further study the balance between immune activation and immune regulation in CVIDid, we used flow cytometry to evaluate naïve T-cell subsets, T-cell activation and cytokine production, exhaustion, negative regulators of immune activation, regulatory T-cells, and T-cell homing markers.

## Methods

### Ethics Statement

Ethical approval for this study for all participants was received from the Medical Ethical Committee of the Erasmus MC University Medical Center in Rotterdam, The Netherlands (METC: 2013–026). Written informed consent was obtained from all patients and controls according to the Declaration of Helsinki.

### Study Population and Sample Collection

Patients were diagnosed with CVID according to the European Society for Immunodeficiencies criteria [1] and were included at the outpatient clinics of the UMC Utrecht and the Erasmus MC University Medical Center Rotterdam, The Netherlands. Patients were eligible if they were aged seven or older, and they and/or their legal guardians signed informed consent. Household members of patients were recruited as healthy controls (HC). Medication use up to 3 months prior to sampling was recorded.

### Sample Processing

Peripheral blood mononuclear cells (PBMC) were isolated from blood by Ficoll density centrifugation (GE Healthcare-Biosciences, AB), and frozen at – 180 °C until use. Cells were subsequently thawed, counted and plated at 1,000,000 live cells per panel per sample. For the panels including intracellular cytokine measurement, cells were first restimulated with 20 ng/mL phorbol 12-myristate 13-acetate (PMA, MilliporeSigma) and 1 μg/mL ionomycin (MilliporeSigma) for 4 h at 37 °C with addition of monensin (Golgistop, BD Biosciences, 1:1500) during the last 3.5 h. Cell death was stained in all panels using Fixable Viability Dye eFluor 506 (eBioscience). Next, cells were incubated with the surface antibodies (Supplementary Table 1) for 20 min at 4 °C and washed. Cells were then permeabilized with fixation/permeabilization reagent (eBioscience) for 30 min at 4 °C, washed, and incubated with the intracellular antibodies (Supplementary Table 1). Cells were stored at 4 °C until the next day, when they were measured on the LSR Fortessa (BD Biosciences).

### Analysis and Statistics

For flow cytometric data, median fluorescence intensities (MFI) and percentages of positive cells were analyzed in FlowJo. All statistical analyses and graphic representations were done in R 3.2.0 [21]. Continuous variables were compared using the Mann–Whitney rank test, or paired Wilcoxon-rank test for paired samples. Correlation was calculated using Spearman’s correlation. P values below 0.05 were considered statistically significant.

## Results

### The Overall T-cell Profile of CVIDid Patients Differs from CVIDio and HC

To investigate immune activation and regulation in CVID, PBMC were isolated from 12 healthy controls (HC), 12 CVID patients with infections only (CVIDio), and 20 patients with CVIDid (Table 1). Patients were selected from a cross-sectional Dutch primary immunodeficiency cohort when they received IgRT and did not use any immunomodulatory medication during and the last 3 months prior to sampling. Three of the CVIDid patients had a known CVID-associated monogenetic disease (CTLA4 haploinsufficiency, STAT1 gain of function, and PIK3R1). Flow cytometry was performed as described in the Supplementary Information (see also Supplementary Table 1 and Supplementary Figs. 1–4).

**Table 1:** Cohort characteristics. *HC*, healthy control; *CVIDio*, CVID with infections only; *CVIDid*, CVID with immune dysregulation; *IQR*, interquartile range; *DMARD*, disease modifying anti-rheumatic drug; *VUS*, variant of unknown significance

Summary statistics	HC (n = 12)	CVIDio (n = 12)	CVIDid (n = 20)
Characteristics			
Age (median, IQR)	45.50 (40.25, 52.00)	38.50 (29.00, 58.25)	38.50 (35.75, 43.50)
Sex (male)	5 (41.67%)	4 (33.33%)	11 (55.00%)
Center (Utrecht)	9 (75.00%)	11 (91.67%)	15 (75.00%)
Antibiotics	0 (0.00%)	3 (25.00%)	8 (40.00%)
Immunosuppressive medication	0 (0.00%)	0 (0.00%)	0 (0.00%)
IgA < 0.1 g/L	0 (0.00%)	2 (16.67%)	13 (65.00%)
Immune dysregulation complications			
Pulmonary	0 (0.00%)	0 (0.00%)	8 (40.00%)
Hematological	0 (0.00%)	0 (0.00%)	2 (10.00%)
Gastrointestinal	0 (0.00%)	0 (0.00%)	8 (40.00%)
Rheumatological	0 (0.00%)	0 (0.00%)	6 (30.00%)
Dermatological	0 (0.00%)	0 (0.00%)	4 (20.00%)
Hematological malignancy	0 (0.00%)	0 (0.00%)	2 (10.00%)
Lymphoproliferation (incl splenomegaly)	0 (0.00%)	0 (0.00%)	10 (50.00%)
Other	0 (0.00%)	0 (0.00%)	4 (20.00%)
DMARD-naïve/subclinical disease	NA	NA	12 (60.00%)
Disease in remission	NA	NA	8 (40.00%)
Genetics			
Not done	12 (100.00%)	12 (100.00%)	12 (60.00%)
Nothing found	0 (0.00%)	0 (0.00%)	2 (10.00%)
VUS found	0 (0.00%)	0 (0.00%)	2 (10.00%)
Pathogenic mutations found	0 (0.00%)	0 (0.00%)	3 (15.00%) + 1 TNFRSF13B (TACI) mutation

**Fig. 1:**
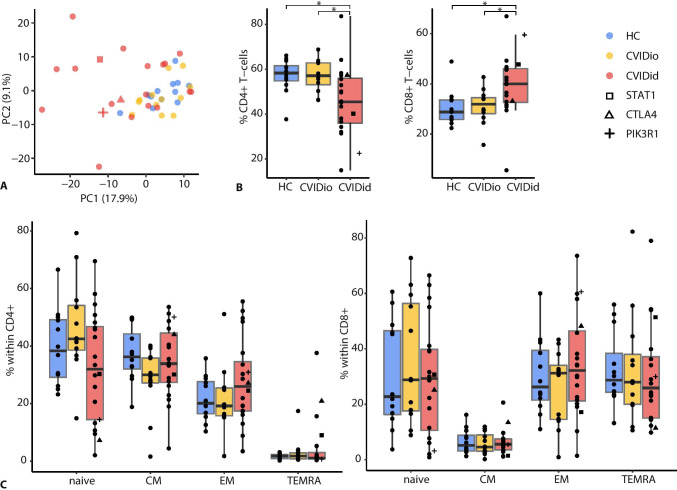
General description of T-cell subsets in CVID. **A** Principal component analysis of FACS data (all panels combined). **B** CD4 + and CD8 + T-cells. **C** Naïve (CD45RA + CCR7 +), central memory (CM: CD45RA − CCR7 +), effector memory (EM: CD45RA − CCR7 −) and terminally differentiated effector memory cells (TEMRA: CD45RA + CCR7 −) in CD4 + T-cells. CVIDid = CVID with immune dysregulation (n = 20), CVIDio = CVID with infections only (n = 12), HC = healthy controls (n = 12). Statistics: Mann–Whitney U-test. *p < 0.05, **p < 0.01, ***p < 0.001

First, pooled flow cytometry data was analyzed in an unsupervised manner using principal component analysis (PCA, Fig. 1A). CVIDio and HC clustered closely together and were distinct from most CVIDid samples. This suggests that most variation in the flow cytometric T-cell data related to immune dysregulation and not to the hypogammaglobulinemia shared by CVID patients. CVIDid patients did not cluster by treatment history or location of autoimmunity (Supplementary Fig. 5), suggesting that peripheral blood T-cell skewing may be generalized among CVIDid patients.

### CVIDid T-Cells Are Th-1-Skewed and Chronically Activated

Next, the distribution of CD4 + and CD8 + T-cell subsets in CVIDid was assessed. Frequencies in CD4 + , CD8 + , and naïve/memory subsets were similar to that reported in previous studies [12, 22]: a decreased CD4/CD8 ratio (Fig. 1B), and a trend of decreased naïve (CD45RA + CCR7 +) CD4 + T-cells in CVIDid compared to CVIDio (p = 0.07) (Fig. 1C). In addition, a subset of CVIDid patients showed a high percentage of CD4 + T effector memory cells, and CD4 + T effector memory cells re-expressing CD45RA (TEMRA), which are associated with chronic activation such as observed in viral infections [23] (Fig. 1C). Within the naïve CD4 + T-cells, CD31 + recent thymic emigrants were more abundant in CVIDid than the CD31-central naïve T-cells (Supplementary Fig. 6). No differences in CD8 + T-cell distribution were observed (Fig. 1C).

**Fig. 2:**
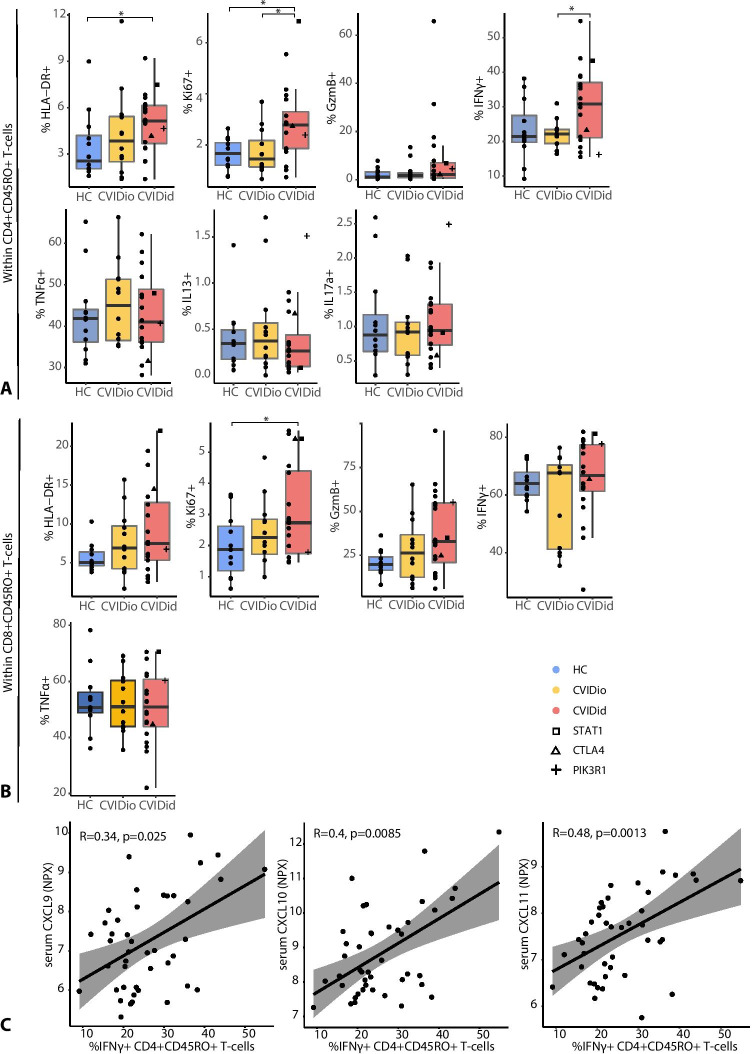
Expression of activation markers HLA-DR, Ki67, and GzmB, and of intracellular cytokines after PMA/ionomycin stimulation: IFNg, TNFa, IL-13, and IL-17a. **A** CD4 + CD45RO + T-cells. **B** CD8 + CD45RO + T-cells. CVIDid = CVID with immune dysregulation (n = 20), CVIDio = CVID with infections only (n = 12), HC = healthy controls (n = 12). Statistics: Mann–Whitney U-test. *p < 0.05, **p < 0.01, ***p < 0.001. **C** %IFNg + CD4 + T-cells correlate with serum levels of CXCL9, CXCL10, and CXCL11. Statistics: Spearman correlation

Within the effector/memory (CD45RO +) subpopulation, proportions of CD4 + T-cells expressing HLA-DR, Ki67, and IFNγ were significantly increased in CVIDid, while IL17a, IL13, and TNF-α expressing T-cells were not different between CVIDid and CVIDio (Fig. 2A). A similar activation pattern was observed in the CD8 + effector memory population for HLA-DR, Ki67, and GzmB (Fig. 2B). We previously described [17] serum cytokine and chemokine levels in an overlapping cohort, allowing comparison between soluble serum markers and T-cell characteristics. Pooling the data from HC, CVIDio, and CVIDid, we observed that the proportion of IFNγ + Th cells correlated with serum levels of interferon-inducible chemokines CXCL9, 10, and 11 (Fig. 2C). While we observed an increase of serum IL17a in CVIDid, there was no corresponding increase of IL17a-producing Th cells, and the frequency of these cells did not correlate with the serum IL17a levels (Supplementary Fig. 7).

### T-cells Expressing Negative Regulators of Immune Activation Retain Their Inflammatory Potential in CVIDid

**Fig. 3:**
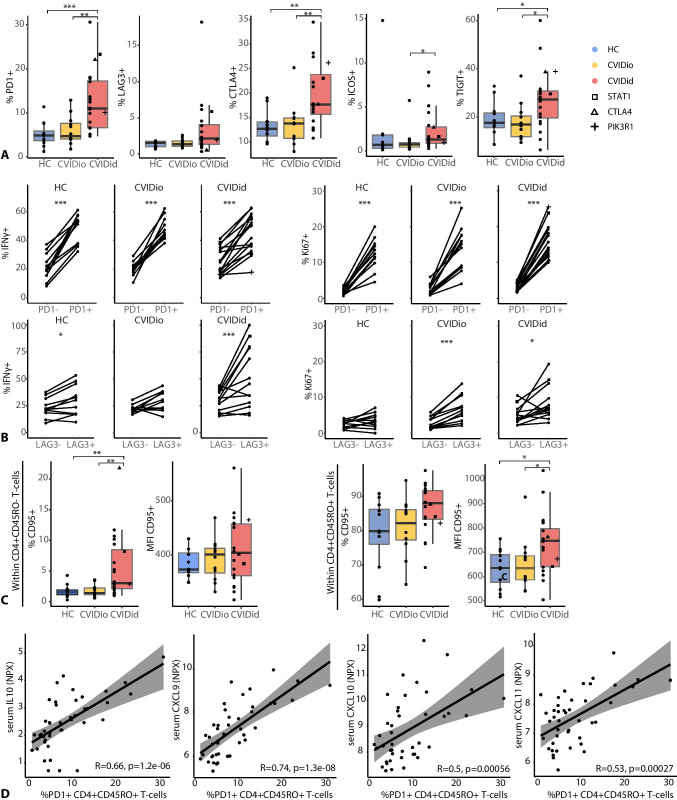
Negative regulators of immune activation in CVID. **A** Proportions of PD1, LAG3, CTLA4, ICOS, and TIGIT in CD4 + CD45RO + T-cells. **B** Percentage and median fluorescence intensity of CD95 (FAS-L) in naïve (CD45RO −) and effector-memory (CD45RO +) CD4 + T-cells. **C** Comparison of IFNγ + and Ki67 + cells in PD1- and LAG3-positive and negative populations. Only samples with > 50 events in the PD1/LAG3-positive and PD1/LAG3-negative populations were included. **D** Spearman correlation between PD1 and IL10, CXCL9, CXCL10, or CXCL11. CVIDid = CVID with immune dysregulation (n = 20), CVIDio = CVID with infections only (n = 12), HC = healthy controls (n = 12). Statistics (A&C): Mann–Whitney U-test. Statistics B: paired Wilcoxon-Rank test. *p < 0.05, **p < 0.01, ***p < 0.001

Next, we investigated whether this immune activation resulted in immune exhaustion in CVIDid, which is known to happen in the context of chronic inflammation [24]. In CVIDid CD4 + -cells, we observed an increased proportion of cells expressing negative regulators of immune activation PD1, LAG3, CTLA4, ICOS, and TIGIT (Fig. 3A). In addition, CVIDid CD4 + T-cells expressed higher levels of CD95 (FAS-L), showing that they are terminally differentiated and may be more prone to apoptosis (Fig. 3B). In CD8 + T-cells, LAG3, CTLA4, ICOS, and TIGIT, but not PD1 and CD95, were similarly increased in CVIDid (Supplementary Fig. 8).

In order to assess whether the PD1 and LAG3 expressing cells were functionally exhausted, production of IFNγ, GzmB, Ki67, and CD95 was compared between the PD1/LAG3 + and PD1/LAG3 − populations (Fig. 3C and Supplementary Fig. 9). In CD4 + T-cells, the PD1 + and LAG3 + subpopulations showed equal or increased expression of IFNγ, Ki67, and/or GzmB as the PD1 − and LAG3 − subpopulations, indicating retained functional capacity. Expression of PD1 correlated with serum levels of pro-inflammatory CXCL9, 10, and 11 as well as immune regulatory IL10 (Fig. 3D), further illustrating the association between PD1 and chronic inflammation. In CD8 + T-cells, IFNγ and GzmB expression was also intact in PD1 + and LAG3 + cells, but Ki67 + cells were less frequent in the LAG3 + subpopulation, indicating reduced proliferative capacity (Supplementary Fig. 10).

### CVIDid Regulatory T-cells Fail to Upregulate CTLA4

**Fig. 4:**
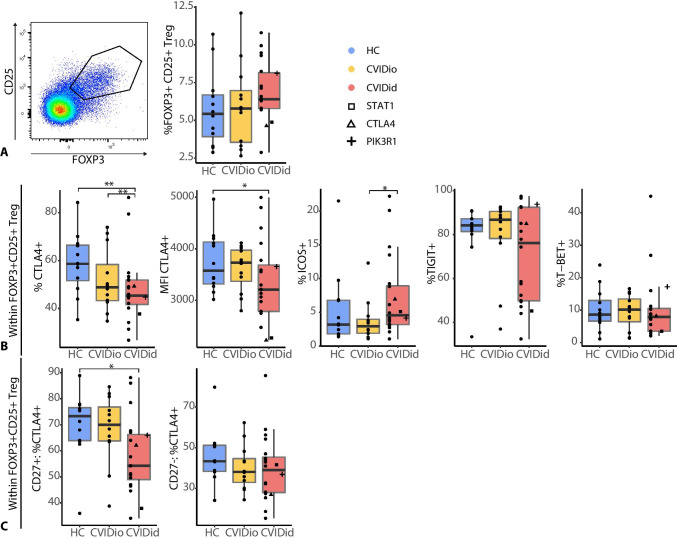
Regulatory T-cells in CVID. **A** Gating strategy and proportion of CD25 + FOXP3 + T-cells within CD4 + population. **B** Expression of CTLA4, ICOS, TIGIT, and T-BET in the Treg population. **C** Decreased fraction of CTLA4 + Tregs was confined to the CD27 + population. CVIDid = CVID with immune dysregulation (n = 20), CVIDio = CVID with infections only (n = 12), HC = healthy controls (n = 12). Statistics: Mann–Whitney U-test. *p < 0.05, **p < 0.01, ***p < 0.001

In addition to negative regulators of immune activation, regulatory T (Treg) cells also contribute to limiting inflammation-related pathology. In contrast to previous studies [25], we did not observe a decreased proportion of CD25 + FOXP3 + Tregs in CVIDid, and a subgroup of CVIDid patients had even increased Treg frequencies (Fig. 4A). ICOS expression in CVIDid Tregs also indicated normal-to-increased activation [26] of Tregs (Fig. 4B), but CTLA4 expression was reduced in CVIDid Tregs, both in cell frequency as MFI in the CTLA4-positive population (Fig. 4B). The failure to upregulate CTLA4 in CVIDid Tregs was isolated to the activated and usually highly suppressive [27] CD27 + Treg population (Fig. 4C), and may result in reduced Treg function, as expression of CTLA4 is important for the suppressive function of Tregs [28]. In addition, a subpopulation of CVIDid patients showed low levels of TIGIT-expressing Tregs, which may impair TIGIT-driven selective suppression of Th1 and Th17 effector cells [29].

### Increased Migratory Capacity in CVIDid T-cells

**Fig. 5:**
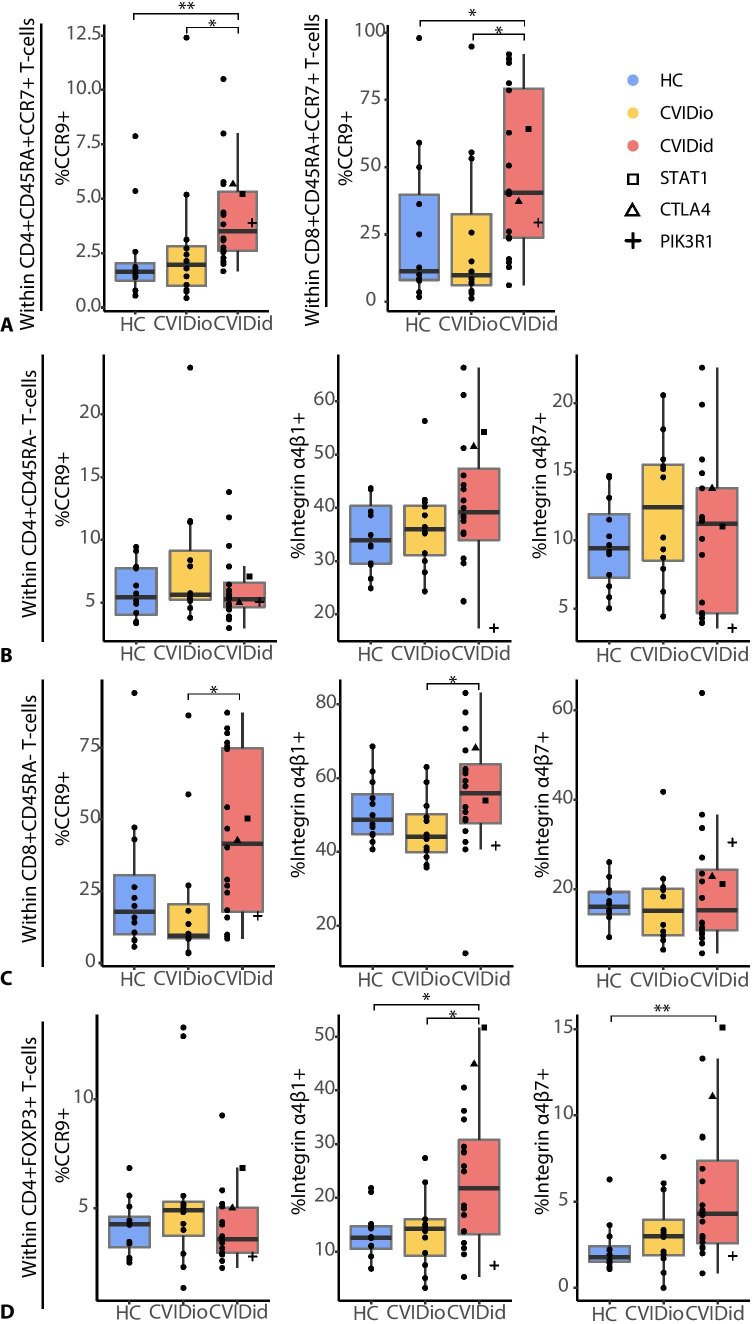
Migratory capacity in CVID: **A** in CCR7 + CD45RA + naïve CD4 and CD8 + T-cells, **B** in non-naïve CD45RA − CD4 + T-cells, **C** in non-naïve CD45RA − CD8 + T-cells, **D** in FOXP3 + CD4 + T-cells. CVIDid = CVID with immune dysregulation (n = 20), CVIDio = CVID with infections only (n = 12), HC = healthy controls (n = 12). Statistics: Mann–Whitney U-test. *p < 0.05, **p < 0.01, ***p < 0.001

The immune dysregulation of most CVIDid patients occurs not only in the systemic immune system, but also locally in affected tissues, which are often mucosal sites (lung and/or gut). Expression of the mucosal homing marker CCR9 was increased on naïve CD4 + and CD8 + populations of CVIDid T-cells (Fig. 5A), but not on non-naïve (CD45RA −) CD4 + T-cells (Fig. 5B), suggesting that this upregulation was at least not entirely antigen-driven. CVIDid CD8 + non-naïve (CD45RA −) T-cells (Fig. 5C) did also show increased expression of CCR9 as well as integrin α4β1, which mediates homing to inflamed tissues, including the lung. CVIDid FOXP3 + CD4 + T-cells (Fig. 5D) also showed increased migratory capacity, as they were enriched for cells expressing integrin α4β1 and gut-homing marker integrin α4β7.

### Patterns of T-cell Dysregulation in CVIDid Patients with GLILD

**Fig. 6:**
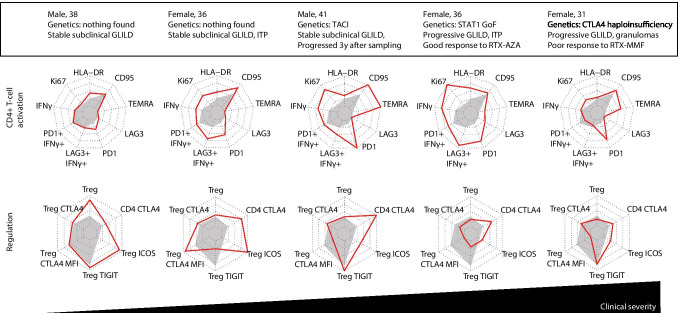
Patterns of CD4 + T-cell activation: % of HLA-DR, Ki67, IFNg, IFNg in the PD1 + population and the LAG3 + population, %PD1, %LAG3, %CD45RA + CCR7 − (TEMRA) cells, and % of CD95. And patterns of T-cell regulation: %CD25 + FOXP3 + Treg, %CTLA4 in Treg, MFI of CTLA4 in Treg, %TIGIT in Treg, %ICOS in Treg, %CTLA4 in CD4 + T-cells in five patients with GLILD. Red line indicates individual patient legends, gray-shaded area indicates the median for the CVIDio group. Axis ranges are the minimum and the maximum for that marker for the entire cohort. MFI, median fluorescence intensity; GLILD, granulomatous-lymphocytic interstitial lung disease; VUS, variant of unknown significance; ITP, idiopathic thrombocytopenia purpura; RTX, rituximab; AZA, azathioprine; MMF, mycophenolate mofetil; SCT, stem-cell transplantation; GOF, gain of function

Finally, in order to illustrate patterns of T-cell immune dysregulation in individual patients with varying severity of specific immune dysregulation phenotypes, we selected markers most strongly associated with (chronic) immune activation in CD4 + T-cells (HLA-DR, Ki67, IFNγ, and IFNγ in PD1 + and LAG3 + , PD1 + , LAG3 + , TEMRA, and CD95) and immune regulation (Tregs, CTLA4 in Tregs, MFI of CTLA4 in Tregs, TIGIT in Tregs, ICOS in Tregs, and CTLA4 in CD4 + T-cells) in CVIDid. We investigated these in five patients with GLILD (Fig. 6), ranging from stably mild radiographic GLILD without lung function deterioration (patients 1 and 2), to clinical GLILD that required immunosuppressive treatment shortly after sampling (patients 4 and 5). In patients 1–4, immune activation increased with severity of disease. In patient 3, who at the time of sampling had subclinical GLILD that exacerbated 3 years later, as well as patients 4 and 5, the median fluorescence intensity of CTLA4 on Tregs decreased, which was not observed for the two subclinical GLILD patients that remained stable (1 and 2). Patient 4 with STAT1 GoF, who required treatment for GLILD shortly after sampling, showed high levels of immune activation and PD1 expression, and low Treg functional markers. Finally, patient 5 with CTLA4 haploinsufficiency demonstrated low CTLA4 expression on Tregs as expected, but also low T-cell activation (HLA-DR, Ki67, and IFNγ) in peripheral blood, despite progressive GLILD and the need for treatment shortly after sampling.

## Discussion

In this study, we showed that T-cells in patients with CVIDid were more often activated, proliferating, and Th1-skewed than those of patients with CVIDio. In addition, more CVIDid T-cells expressed immune co-inhibitory receptors PD1, LAG3, CTLA4, ICOS, and TIGIT, and these cells retained their inflammatory properties. Chronic activation was observed in both the CD4 + and CD8 + compartment. In the Treg compartment, we observed low CTLA4 expression in CVIDid, while ICOS expression remained intact. Finally, CVIDid T-cells showed increased migratory capacities to mucosal tissues.

High T-cell activation and Th-1 skewing in CVIDid were consistent with previous studies [12, 18]. In addition, we did not observe increased frequencies of IL17-producing T-cells, despite our previous finding of increased IL-17a in serum of the same patients sampled at the same time. This supports the hypothesis for an alternative source of IL17a production in CVIDid, such as type-3 ILCs [30].

Previous studies have also reported increased PD1 expression in CVID, and interpreted this as a sign of functional exhaustion and impaired T-cell function [15, 18, 31]. However, we observed that the PD1- and LAG3-expressing cells in CVIDid retained the capacity to produce pro-inflammatory cytokines and to proliferate, and thus were not functionally exhausted. Upregulation of negative regulators of co-stimulation has been suggested to be a mechanism to limit inflammation-related damage to tissues in settings of chronic inflammation, while maintaining the ability to respond to pathogens [32]. In CVIDid, however, it is possible that this compensatory response is insufficient in severe states of immune dysregulation and that these chronically activated cells still contribute to immune dysregulation-related pathology.

In addition to this chronically activated T-cell state, we observed a decreased ability of Tregs to upregulate CTLA4, while CTLA4 expression was increased in the whole CD4 + population. Expression of CTLA4 by Tregs is an important mechanism by which Tregs mediate their suppressive function [33]. Clinical CTLA4 haploinsufficiency often results in a CVIDid phenotype with hypogammaglobulinemia and autoimmune disease, and functional Treg dysfunction has been described [19]. In this study, the T-cell profile of the patient with CTLA4 haploinsufficiency was often not very different from the other non-genetic CVIDid patients. Therefore, the expression of CTLA4 in Tregs of non-genetic CVIDid patients may be relevant to the overall underlying pathophysiology of CVIDid and warrants further research. A recent study shows that abatacept, a CTLA4 fusion protein, was safe and effective in the treatment of CVIDid with interstitial lung disease [34]. As the population of CVIDid patients with low CTLA4 + CD27 + Tregs represented a mix of patients with pulmonary inflammation but also other organ-specific autoimmunity (data not shown), abatacept may be efficacious in other CVIDid patients as well. In addition, longitudinal monitoring of CTLA4 Treg expression in CVIDid may indicate whether it can be used as a biomarker for disease exacerbation and/or therapeutic response.

Despite these overall differences between CVIDid and CVIDio, the heterogeneity within the CVIDid group was substantial. Subgroup analyses of patients with organ-specific autoimmunity did not yield insightful patterns, except that GLILD patients were often more extreme in all observed differences (data not shown). In addition, disease severity did not always reflect immune activation. For example, the patient with CTLA4 haploinsufficiency did not show an inflammatory state in peripheral blood, while the patient that had the strongest IFNγ signature combined with low immune regulation markers (Supplementary Fig. 11) was clinically stable and did not require immunosuppressive therapy. It is possible that the peripheral blood immune phenotype only gives limited information that is clinically relevant, as T-cells may have migrated to the inflamed tissues in the sickest patients. In addition, this study may be limited by sample size to detect differences between subgroups of CVIDid. One other aspect that this study does not address is differences in absolute T-cell numbers. A recent study showed that while absolute T-cells are often lower in CVID, they do not differ between CVIDio and CVIDid [13].

To conclude, these results indicate that CVIDid Th cells are highly activated, and that, unlike in classically exhausted cells such as originally described in chronic viral infection and cancer [32], PD1 and LAG3 expression in CVIDid CD4 + T-cells reflects chronic activation with preserved inflammatory potential rather than functional exhaustion, similar to previous findings in human auto-immune inflammation [24]. Combined studies of T-cell dysfunction and circulating inflammatory proteins in peripheral blood may help predict response to T-cell-targeted therapies in individual CVIDid patients. Moreover, loss of CTLA4 upregulation in activated CVIDid Tregs may be mechanistically important in maintaining the inflammatory loop in CVIDid, and warrants further research.

## Supplementary Information

Below is the link to the electronic supplementary material.

Supplementary File 1 (DOCX 1.04 MB)

## Data Availability

Not applicable.
