# Supplementary material for: Chronically Activated T-cells Retain Their Inflammatory Properties in Common Variable Immunodeficiency
Source: J Clin Immunol. 2021 Jul 11;41(7):1621–32. doi: 10.1007/s10875-021-01084-6 (PMC8452589; doi:10.1007/s10875-021-01084-6)
Supplement: Supplementary file 1 — (DOCX 1.04 MB) [file 10875_2021_1084_MOESM1_ESM.docx]

**Supplementary Figures**


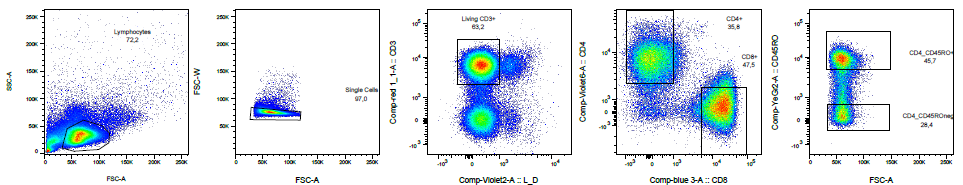


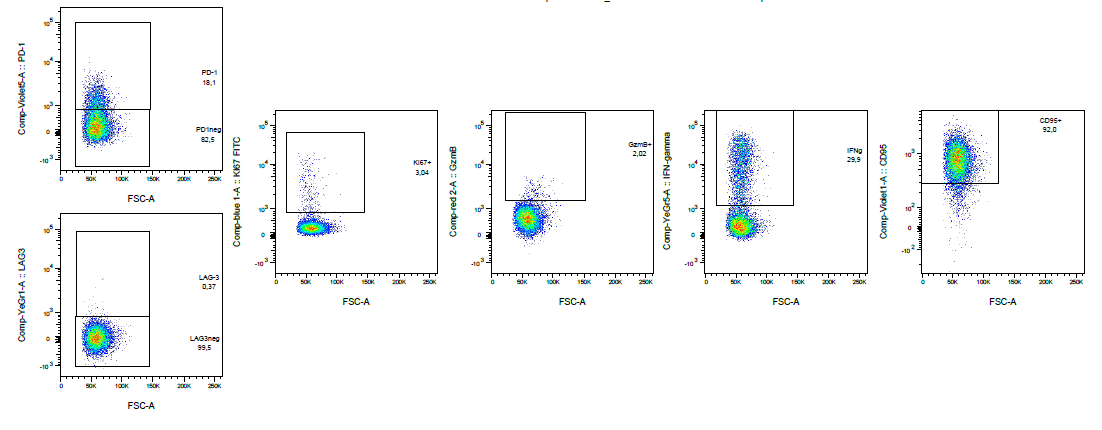


**Supplementary Figure 1**: Representative gating: general gating strategy and exhaustion panel


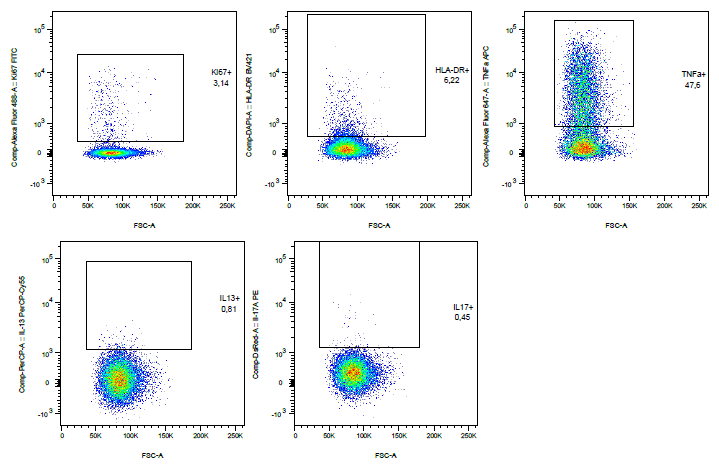


**Supplementary Figure 2**: Representative gating: gating strategy Th skewing panel


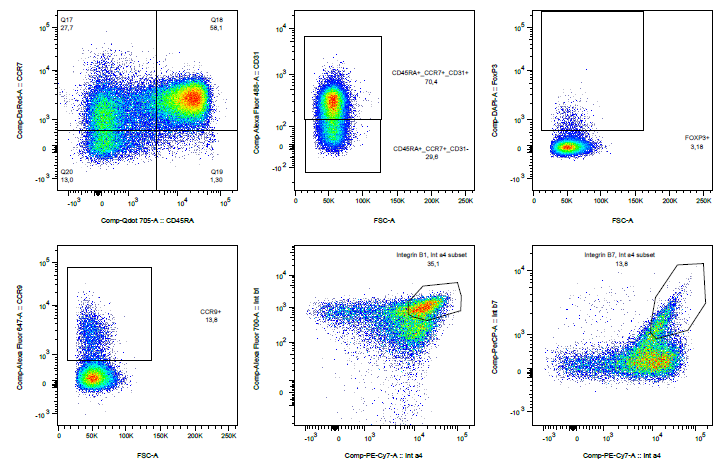


**Supplementary Figure 3**: Representative gating naïve / homing panel


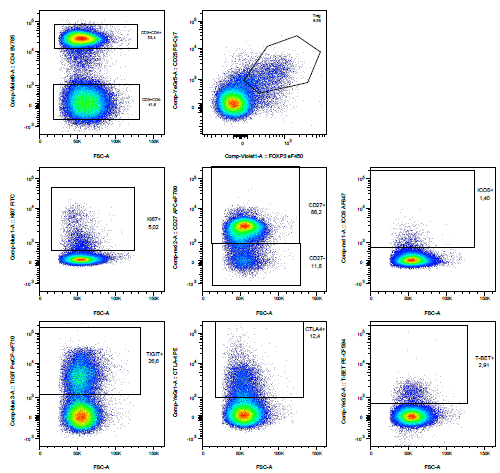


**Supplementary Figure 4**: Representative gating Treg panel


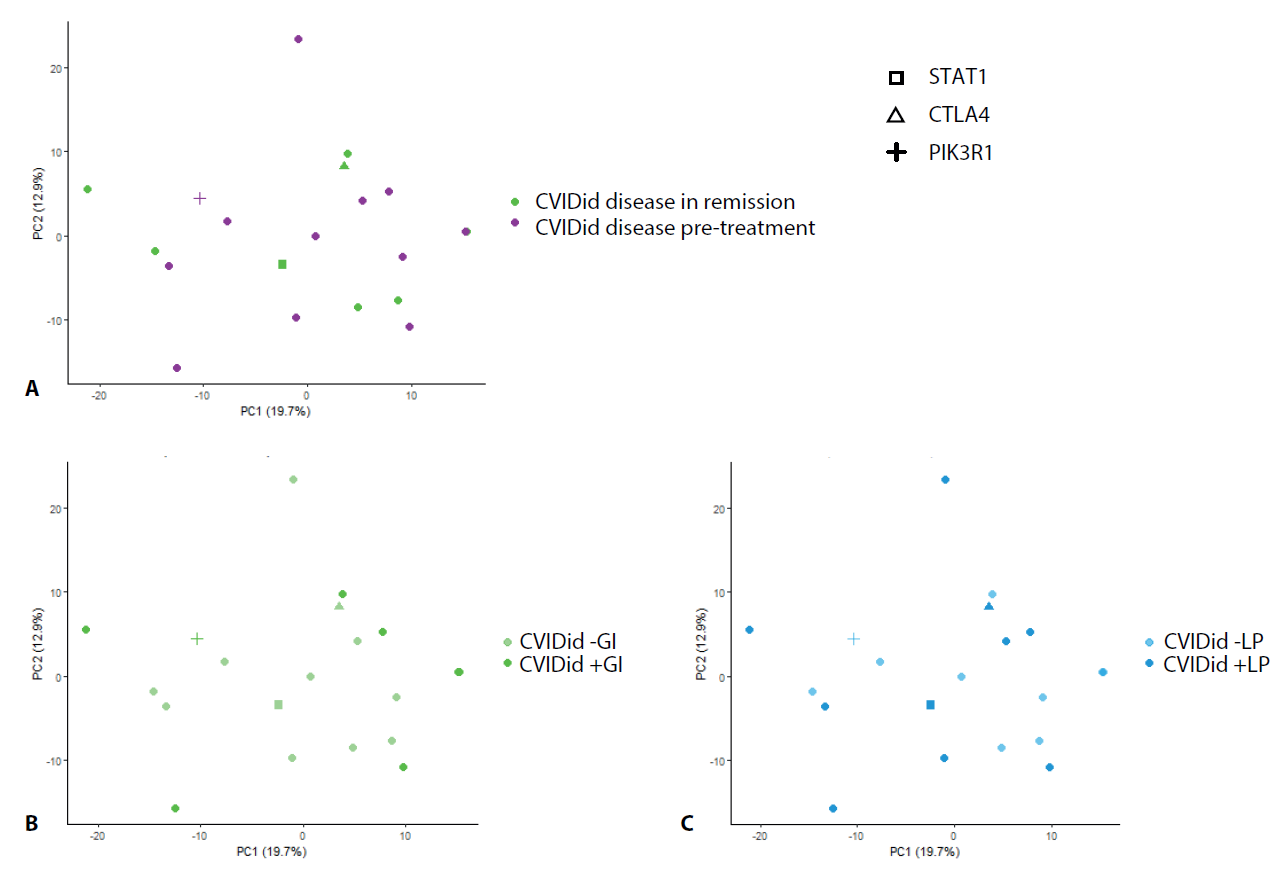


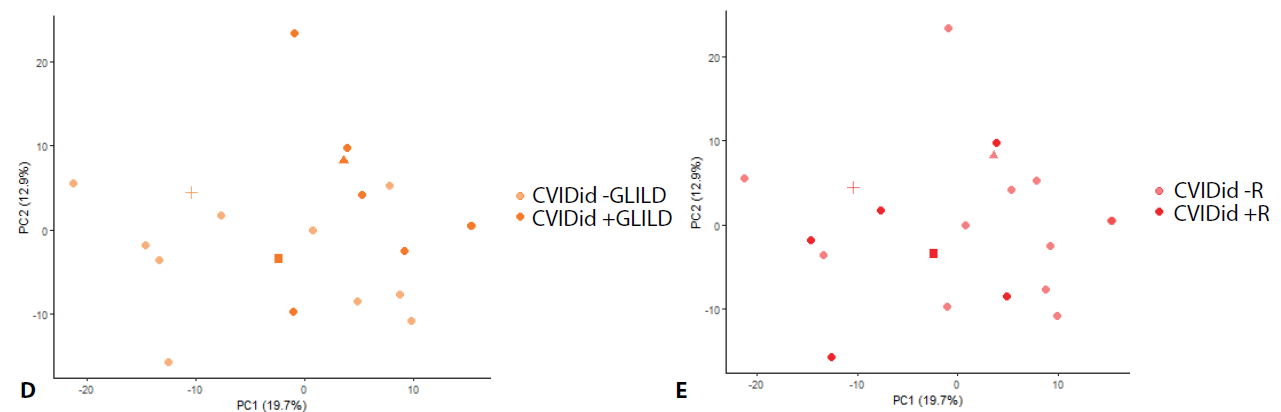


**Supplementary Figure 5**: Principal component analysis of FACS data (all panels combined). CVID with immune dysregulation (CVIDid, n=20) only.

A: disease status: disease in remission (n=8) versus disease pre-treatment (n=12).

B: CVIDid patients with (+GI, n=8) and without (-GI, n=12) gastrointestinal complications.

C: CVIDid patients with (+LP, n=10) and without (-LP, n=10) lymphoproliferation.

D: CVIDid patients with (+GLILD, n=8) and without (-GLILD, n=12) granulomatous-lymphocytic interstitial lung disease

E: CVIDid patients with (+R, n=6) and without (-R, n=14) rheumatological complications.


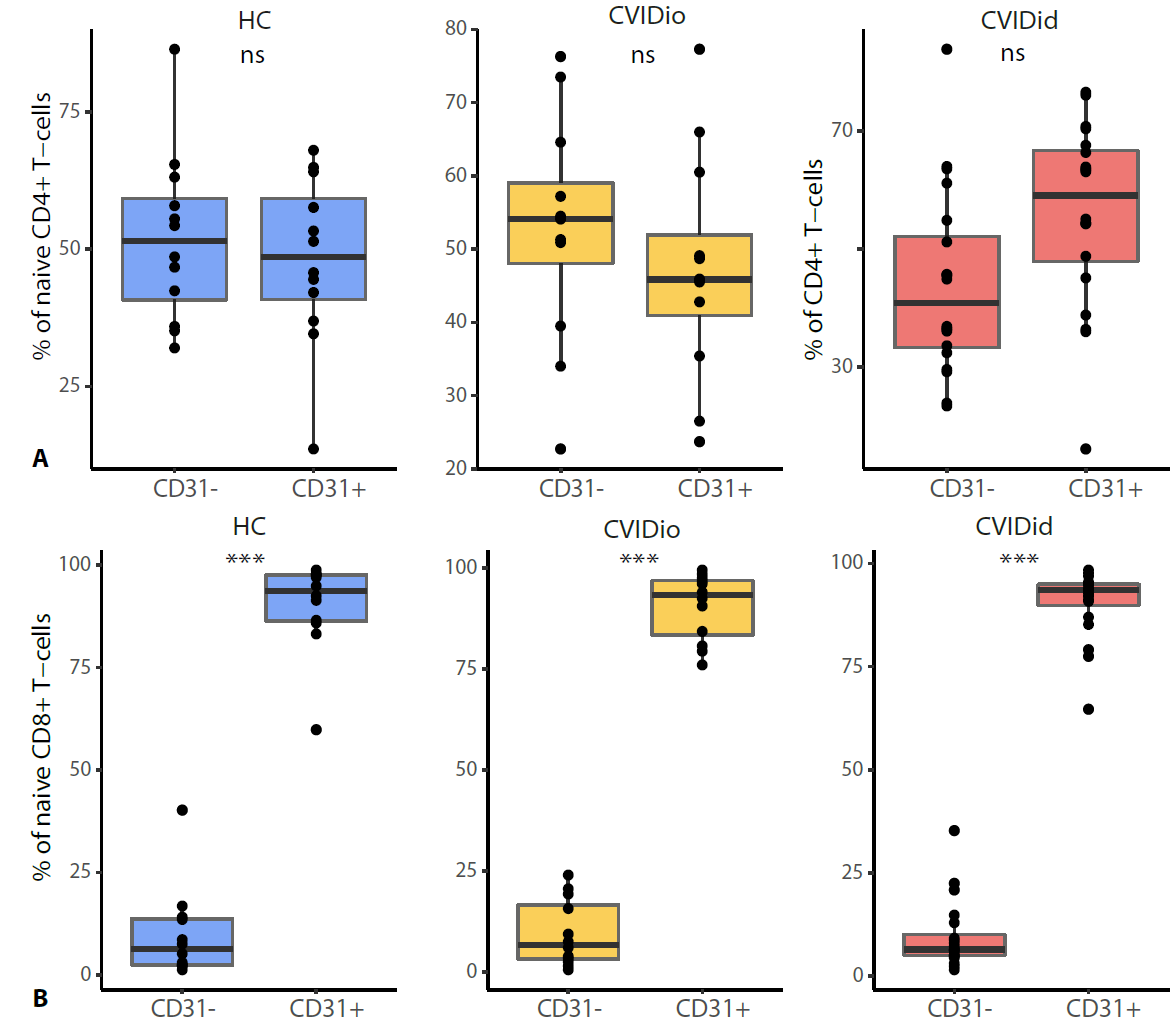


**Supplementary Figure 6**: CD31 expression within naïve (CD45RA+CCR7+) T-cells. CVIDid = CVID with immune dysregulation (n=20), CVIDio = CVID with infections only (n=12), HC = healthy controls (n=12). Statistics: Mann-Whitney U-test. * p<0.05, ** p<0.01, *** p<0.001.

A: naïve CD4+ T-cells

B: naïve CD8+ T-cells


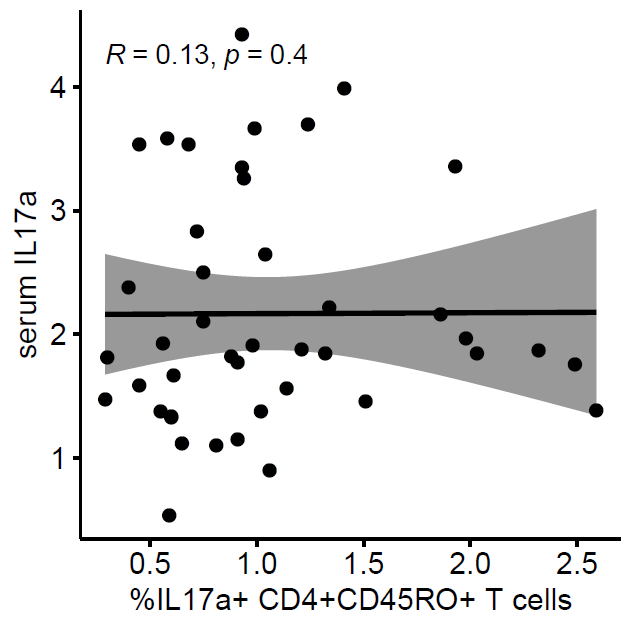


**Supplementary Figure 7**: Percentage of IL17+ CD4+CD45RO+ T-cells did not correlate with serum levels of IL-17A. Statistics: Spearman correlation.


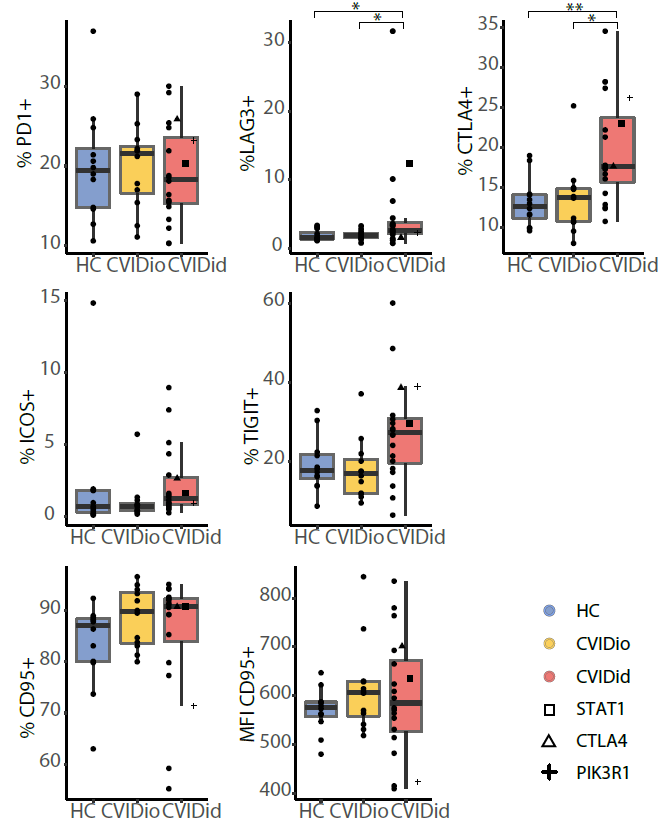


**Supplementary Figure 8**: Proportions of negative regulators of immune activation PD1, LAG3, CTLA4, ICOS and TIGIT, and apoptosis marker CD95 (FASL) in CD8+CD45RO+ T-cells. CVIDid = CVID with immune dysregulation (n=20), CVIDio = CVID with infections only (n=12), HC = healthy controls (n=12). MFI: median fluorescence intensity. Statistics: Mann-Whitney U-test. * p<0.05, ** p<0.01, *** p<0.001.


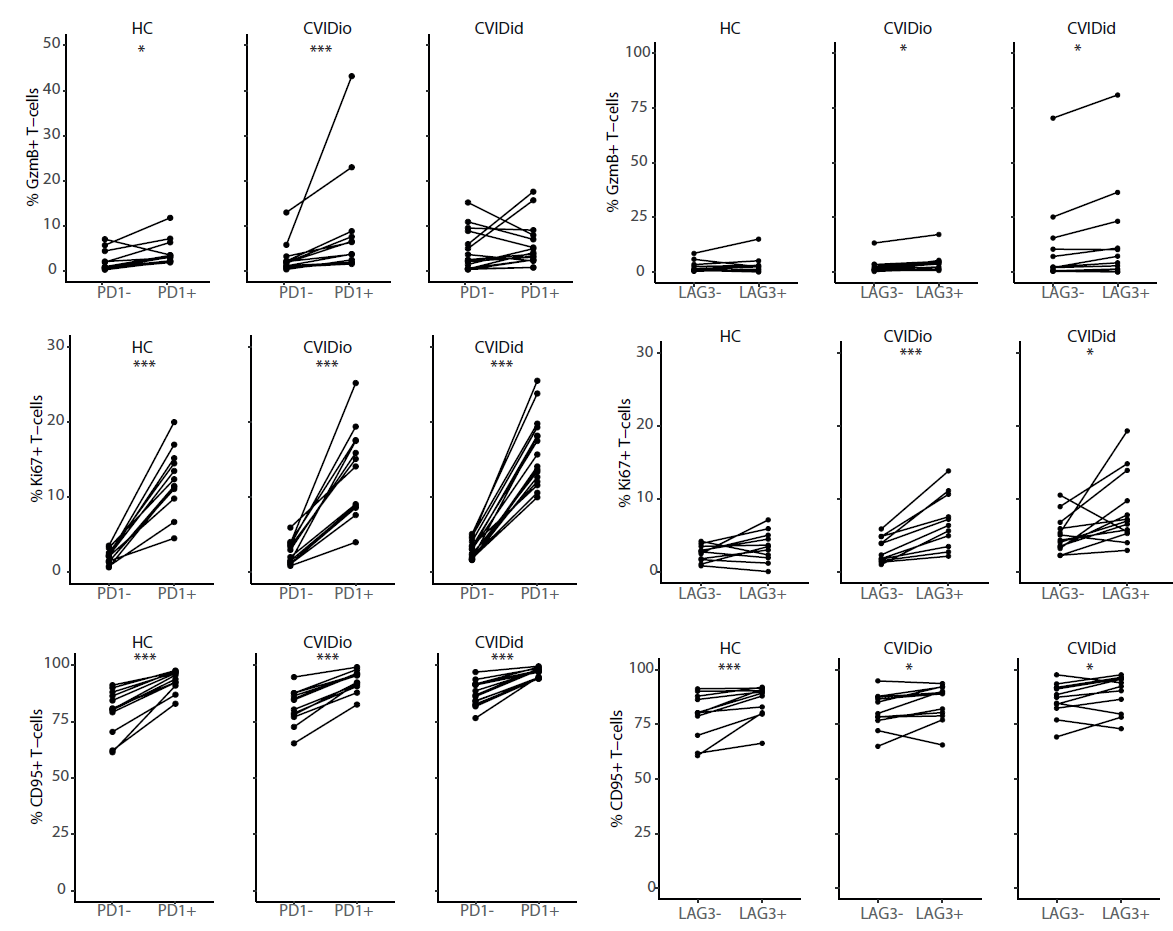


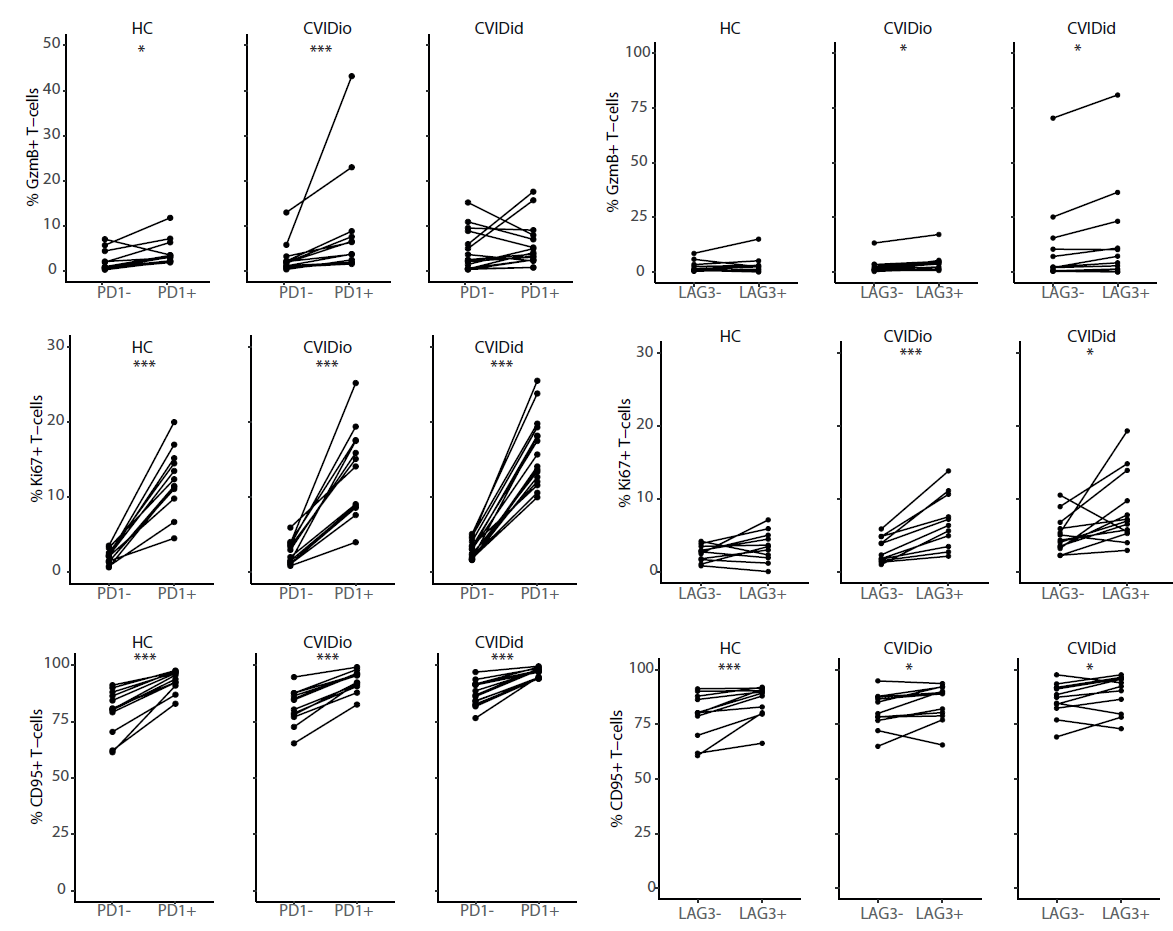


**Supplementary Figure 9**: Comparison of percentage of GzmB+ and CD95+ cells in PD1 and LAG3 positive and negative populations in CD4+CD45RO+ T-cells. Only samples with >50 events in the PD1/LAG3 positive and PD1/LAG3 negative populations were included. CVIDid = CVID with immune dysregulation (n=20), CVIDio = CVID with infections only (n=12), HC = healthy controls (n=12). Statistics: paired Wilcoxon-Rank test. * p<0.05, ** p<0.01, *** p<0.001.


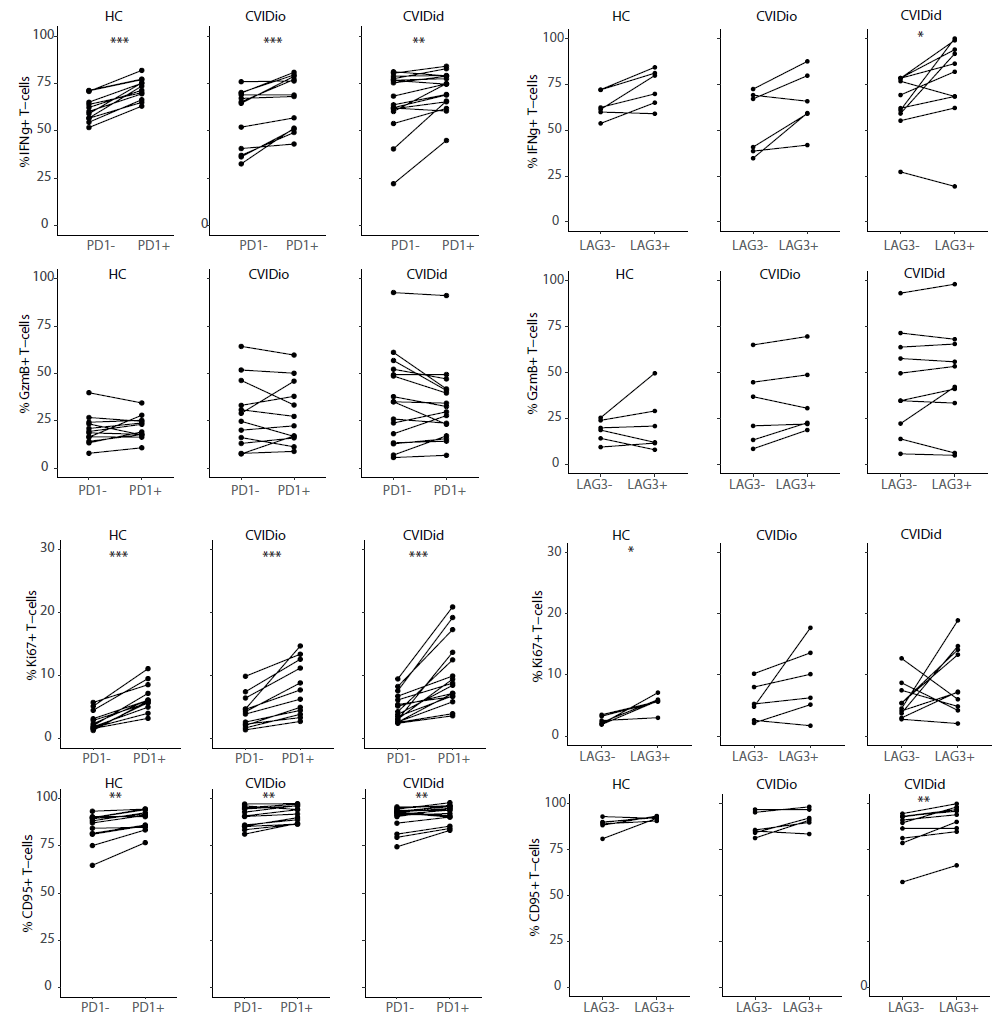


**Supplementary Figure 10**: Comparison of percentage of IFNγ+, GzmB+, Ki67+ and CD95+ cells in PD1 and LAG3 positive and negative populations in CD8+CD45RO+ T-cells. Only samples with >50 events in the PD1/LAG3 positive and PD1/LAG3 negative populations were included. CVIDid = CVID with immune dysregulation (n=20), CVIDio = CVID with infections only (n=12), HC = healthy controls (n=12). Statistics: paired Wilcoxon-Rank test. * p<0.05, ** p<0.01, *** p<0.001.


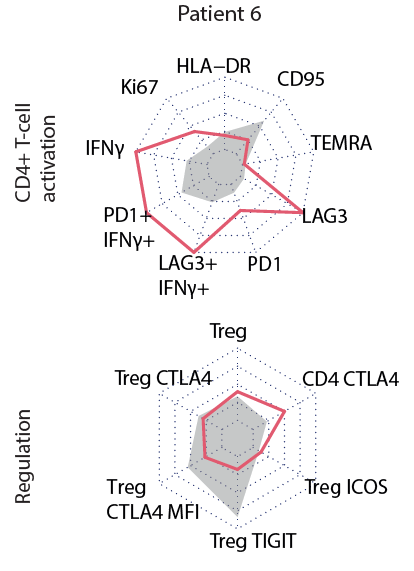


**Supplementary Figure 11**: Patterns of CD4+ T-cell activation and T-cell regulation in a patient with high immune activation markers but mild clinical phenotype. Coeliac disease, Sjögren-like disease, autoimmune gastritis, not requiring immunosuppressive therapy. Genetics were done but no relevant mutations were found. MFI: median fluorescence intensity.

**Supplementary Table 1:** FACS staining antibody list

| **Antibody** | **Fluorochrome** | **Company** | **Panel** |
| --- | --- | --- | --- |
| *Surface* |  |  |  |
| CCR7 | PE | eBioscience | Homing/naive |
| CCR9 | APC | Biolegend | Homing/naive |
| CD137 (4-1BB) | APC | BD | Exhaustion |
| CD25 | PE-Cy7 | BD | Treg |
| CD27 | APC-eF780 | eBioscience | Treg |
| CD3 | AF700 | Biolegend | Exhaustion, Treg |
| CD3 | BV605 | Biolegend | Th skewing, Homing/Naive |
| CD31 | FITC | BD | Homing/naive |
| CD4 | BV785 | Biolegend | Exhaustion, Th skewing, Homing/Naive Treg |
| CD45RA | BV711 | Biolegend | Homing/naive |
| CD45RO | ECD | Beckman Coulter | Exhaustion, Th skewing |
| CD8 | APC-Cy7 | BD | Th skewing, Homing/Naive |
| CD8a | PerCP-Cy5.5 | Biolegend | Exhaustion |
| CD95 (FAS ligand) | eFluor450 | eBioscience | Exhaustion |
| HLA-DR | BV421 | Biolegend | Th skewing |
| ICOS | AF647 | eBioscience | Treg |
| Int α4 | PE-Cy7 | Biolegend | Homing/naive |
| Int β1 | AF700 | Biolegend | Homing/naive |
| Int β7 | PerCP-Cy5.5 | Biolegend | Homing/naive |
| LAG-3 | PE | R&D Systems | Exhaustion |
| PD-1 | BV711 | BD | Exhaustion |
| TIGIT | PerCP-eF710 | eBioscience | Treg |
| TNF | APC | Biolegend | Th skewing |
| *Intracellular* |  |  |  |
| CTLA-4 | PE | BD | Treg |
| FOXP3 | eF450 | eBioscience | Homing/naive, Treg |
| GzmB | APC-Fire750 | Biolegend | Exhaustion |
| IFNy | PE-Cy7 | BD | Exhaustion |
| IL-13 | PerCP-Cy5.5 | Sony Biotechnology | Th skewing |
| IL-17A | PE | eBioscience | Th skewing |
| Ki67 | FITC | Dako (Agilent) | Exhaustion, Th skewing, Treg |
| T-BET | PE-CF594 | BD | Treg |
